# Socioeconomic deprivation and survival outcomes in primary central nervous system lymphomas

**DOI:** 10.3389/fonc.2022.929585

**Published:** 2022-08-26

**Authors:** Xiangyang Deng, Xionggang Yang, Chunlei Yang, Kezhu Chen, Junwei Ren, Jun Zeng, Quan Zhang, Tianwen Li, Qisheng Tang, Jianhong Zhu

**Affiliations:** ^1^ Department of Neurosurgery, Huashan Hospital, Shanghai Medical College, Fudan University, National Center for Neurological Disorders, National Key Lab. for Medical Neurobiology, Institutes of Brain Science, Shanghai Key Lab. of Brain Function and Regeneration, Institute of Neurosurgery, MOE Frontiers Center for Brain Science, Shanghai, China; ^2^ Department of Orthopaedics Surgery, Fudan University Huashan Hospital, Shanghai, China

**Keywords:** socioeconomic deprivation, primary central nervous system lymphoma, SEER, overall survival (OS), cancer-specific survival

## Abstract

**Objective:**

To our knowledge, the impact of area-level socioeconomic status (SES) has not yet been described in primary central nervous system lymphomas (PCNSLs). Current study sought to explore the association of socioeconomic deprivation, measured using the Area Deprivation Index (ADI), with PCNSL outcomes.

**Methods:**

The Surveillance, Epidemiology, and End Results (SEER) database was used to identify PCNSL patients diagnosed between 2006 and 2015 for our analyses. The impact of ADI on overall survival (OS) and cancer-specific survival (CSS) were investigated. Survival analyses were conducted using Kaplan-Meier method with log-rank tests. The Inverse Probability Weighting (IPW) analysis and multivariate cox proportional hazards regression analysis were employed to make covariate adjustments. Multiple mediation analysis (MMA) was performed to estimate the mediating effects.

**Results:**

A total of 3159 PCNSL patients classified into low and high ADI subgroups according to the median ADI score were studied. The Kaplan-Meier analyses showed that low ADI was significantly associated with higher OS rates (HR 1.15, 95%CI 1.06-1.26, P<0.01) and CSS rates (HR 1.15, 95%CI 1.05-1.27, P<0.01). Similar results were observed in analyses adjusted *via* IPW and multivariate cox methods. Subgroup analyses revealed that ADI could remain a prognostic indictor among different subsets. MMA revealed that several factors including chemotherapy and HIV status making up about 40% of the overall effect, mediated PCNSL survival disparities related to the ADI. Finally, multivariable logistic regression analysis showed that ADI as well as several other factors were independently related to receipt of chemotherapy.

**Conclusions:**

Our study highlights the role of area-level SES in prognosis of PCNSLs. And several factors including chemotherapy and HIV status of PCNSL patents contributed to the CSS disparities between ADI subgroups were uncovered by MMA. Such relationships would highlight the importance of policies development to enhance healthcare delivery and promote awareness of HIV prevention and treatment in low-resource neighborhoods.

## Introduction

Primary central nervous system lymphoma (PCNSL) is a type of highly aggressive extranodal non-Hodgkin lymphoma that confined to the central nervous system (CNS) including leptomeninges, brain, eyes, or spinal cord without evidence of systemic disease (1–4). Immunosuppressive patients have a higher risk of developing this disease (5, 6). Historically, PCNSL has carried a poor prognosis with a 5-year survival of only 15-30% (1, 7). Regarding therapeutic measures, high-dose methotrexate-based systemic chemotherapy is identified as the standard first-line treatment (3, 4). Over the past decades, major progresses have been achieved in the treatment for this disease, however, significant disparities in PCNSL outcomes persist.

Among most tumors, black patients usually tend to have worse outcomes than white patients. And increasing research have shown many important clinical differences between cancer patients with different races and ethnicities (8–10). Also, socioeconomic status (SES), as measured by the state of income, wealth, education, occupation, and living conditions, has been found to be associated with cancer survival (11, 12). In PCNSL, few studies have examined the association of SES with disease outcomes. A previous study of PCNSL found that treatment selection in elderly patients was significantly influenced by sex, facility type, degree of urbanization, and type of insurance (13). Another observational study reported that lack of private insurance and residence in poorer areas were significantly associated with the worse outcomes in PCNSL patients (14). Many population-based studies using large database also have demonstrated that area-based SES was an important risk factor for worse prognosis across a variety of tumors (15, 16). However, these researches have tended to employ single-domain SES measures (income, education, poverty, etc.) or create overly simplistic composite neighborhood SES measures (16–19), which had been questioned as underexamined (20), thereby making inaccurate assessment. As a metrics of socioeconomic deprivation, the area deprivation index (ADI) integrates 17 measures of education, employment, housing quality, and poverty based on the long-form US Census data (21, 22); and it can be used to better assess PCNSL prognosis in the context of neighborhood socioeconomic disadvantage.

Therefore, using the Surveillance, Epidemiology, and End Results (SEER) database, we investigated the association of ADI with PCNSL outcomes to gain better insight into the impact of socioeconomic inequality. In-depth understanding the relation between socioeconomic deprivation and cancer prognosis may support policies for ongoing investments in lower-resource neighborhoods, thereby reducing health disparities.

## Methods

### Study population

We used the SEER database to extract research data. Patients diagnosed with PCNSL from 2006 to 2015 were obtained for our analyses. PCNSL patients were identified according to the International Classification of Diseases for Oncology Third Edition (ICD-O-3) histology codes (9590–9599, 9670–9699, 9700–9719, 9720–9729) with the location limited to the central nervous system, as demonstrated in our previous article (23). PCNSLs included in our analysis were restricted to primary cancers and patients diagnosed without histological confirmation or diagnosed by autopsy were excluded.

### Area deprivation index and covariates

As a comprehensive composite measure of neighborhood SES, ADI can be used for county-level SES estimation. Based on the American Community Survey (ACS) 5-year estimates (2006 to 2010 and 2011 to 2015), we employed “sociome” package to calculate the ADI of each patient according to the patient’s five-digit geographic identifiers (24). And all patients were assigned into low- and high-group based on the median ADI score for further research. Data regarding patient demographics (age at diagnosis, sex, race, marital status, insurance status), tumor characteristics (tumor location and tumor histology types), treatment information (surgery type, radiotherapy and chemotherapy) and follow-up time were also extracted from SEER database. The primary endpoints of this study were overall survival (OS) and cancer-specific survival (CSS).

### Statistical analysis

The χ2 tests were employed to compare distributions of categorical covariates stratified by ADI level among PCNSL patients. Survival analyses were conducted using Kaplan-Meier method with log-rank tests. Multivariate Cox proportional hazards regression analyses were employed to make covariate adjustments. For further results enhancement, the Inverse Probability Weighting (IPW) analysis was performed *via* a propensity model to adjust for imbalances by ADI (25). The absolute Standardized Mean Differences (SMDs) were calculated to verify the covariate balances after the IPW adjustment; and a difference of SMD equal to zero indicates ideal balance.

Subgroup analyses were conducted to examine the robustness of ADI effect. Furthermore, we used multiple mediation analysis (MMA) which proposed by Yu et al. to explore how much effect from multiple mediators/confounders involving in the ADI disparity on cancer-specific survival (26). We proposed a mediation model to identify the presence and relative contributions of factors that are influenced by the independent variable (ADI) and that may exert indirect effects on the PCNSL survival. Direct effect and indirect effect were estimated *via* MMA using “mma” package. Indirect effect is the different cancer-specific survival between low and high ADI groups that can be accounted for by selected mediators, while the direct effect is opposite. The purpose of this approach is to explore the existence and feasibility of identifying processes that mediate known ADI disparity. We hypothesize that the relationships between low-SES neighborhood and poor PCNSL outcomes are partially mediated/moderated by treatment or other factors. And by revealing such internal relationships, it would benefit the development of targeted policies for low-SES neighborhood. Finally, we performed multivariable logistic regression analysis to further evaluate the association between clinical and sociodemographic factors and chemotherapy use.

The R software version 4.0.5 (R Foundation for Statistical Computing, Vienna, Austria) was used for all statistical analyses and a two-sided P value of <0.05 indicated statistically significant.

## Results

### Population characteristics

A total of 3159 PCNSL patients met the study’s eligibility criteria, with 1580 cases identified as low ADI patients and 1579 identified as high ADI patients, were further studied. The sociodemographic, clinical characteristics, as well as treatment information of the included patients are summarized in **Table 1**. Overall, most patients (61.3%) were 60 years old or older, about half of patients (53.2%) were male, and 78.6% were white. 42.2% of the tumors were supratentorial and DLBCL (78.7%) was the most common subtype. In terms of treatment measures, majority of patients (69.9%) received chemotherapy, whereas only 23.3% underwent surgical excision and 30.1% received radiotherapy. Additionally, the vast majority of patients were HIV negative. Comparison of patient characteristics between ADI subgroups showed that high ADI was significantly associated with black race, negative HIV status and no chemotherapy implement.

**Table 1:** Patient characteristics stratified by ADI.

α	Overall	Low ADI	High ADI	*P*
Characteristics	(N = 3159)	(N = 1580)	(N = 1579)	
Age				
<60y	1224 (38.7%)	601 (38.0%)	623 (39.5%)	0.435
≥60y	1935 (61.3%)	979 (62.0%)	956 (60.5%)	
Sex				
Male	1680 (53.2%)	835 (52.8%)	845 (53.5%)	0.734
Female	1479 (46.8%)	745 (47.2%)	734 (46.5%)	
Race				
White	2484 (78.6%)	1229 (77.8%)	1255 (79.5%)	<0.01
Black	272 (8.6%)	87 (5.5%)	185 (11.7%)	
AIAN/API	387 (12.3%)	256 (16.2%)	131 (8.3%)	
Unknown	16 (0.5%)	8 (0.5%)	8 (0.5%)	
Tumor site				
Supratentorial	1332 (42.2%)	655 (41.5%)	677 (42.9%)	0.716
Infratentorial	184 (5.8%)	97 (6.1%)	87 (5.5%)	
Spinal cord	176 (5.6%)	85 (5.4%)	91 (5.8%)	
Other/brain, NOS	1467 (46.4%)	743 (47.0%)	724 (45.9%)	
Histology				
DLBCL	2485 (78.7%)	1259 (79.7%)	1226 (77.6%)	0.175
Others	674 (21.3%)	321 (20.3%)	353 (22.4%)	
Surgery type				
No surgery/biopsy	2386(75.5%)	1208(76.5%)	1178(74.6%)	<0.01
STR	264 (8.4%)	135 (8.5%)	129 (8.2%)	
GTR	472 (14.9%)	224 (14.2%)	248 (15.7%)	
Unknown	37 (1.2%)	13 (0.8%)	24 (1.5%)	
Radiotherapy				
No	2208 (69.9%)	1127 (71.3%)	1081 (68.5%)	0.086
Yes	951 (30.1%)	453 (28.7%)	498 (31.5%)	
Chemotherapy				
No	950 (30.1%)	429 (27.2%)	521 (33.0%)	<0.01
Yes	2209 (69.9%)	1151 (72.8%)	1058 (67.0%)	
Marital status				
Single	1246 (39.4%)	594 (37.6%)	652 (41.3%)	0.101
Married	1790 (56.7%)	924 (58.5%)	866 (54.8%)	
Unknown	123 (3.9%)	62 (3.9%)	61 (3.9%)	
HIV status				
Negative/unknown	2998 (94.9%)	1520 (96.2%)	1478 (93.6%)	<0.01
Positive	161 (5.1%)	60 (3.8%)	101 (6.4%)	
Insurance status				
No	113 (3.6%)	47 (3.0%)	66 (4.2%)	0.155
Yes	2665 (84.4%)	1347 (85.3%)	1318 (83.5%)	
Unknown	381 (12.1%)	186 (11.8%)	195 (12.3%)	

AIAN/API, American Indian/Alaska Native or Asian Pacific Islander; DLBCL, diffuse large B-cell lymphoma; NOS, not otherwise specified; STR, subtotal resection; GTR, gross total resection; ADI, area deprivation index.

### Survival analysis

**Figure 1:**
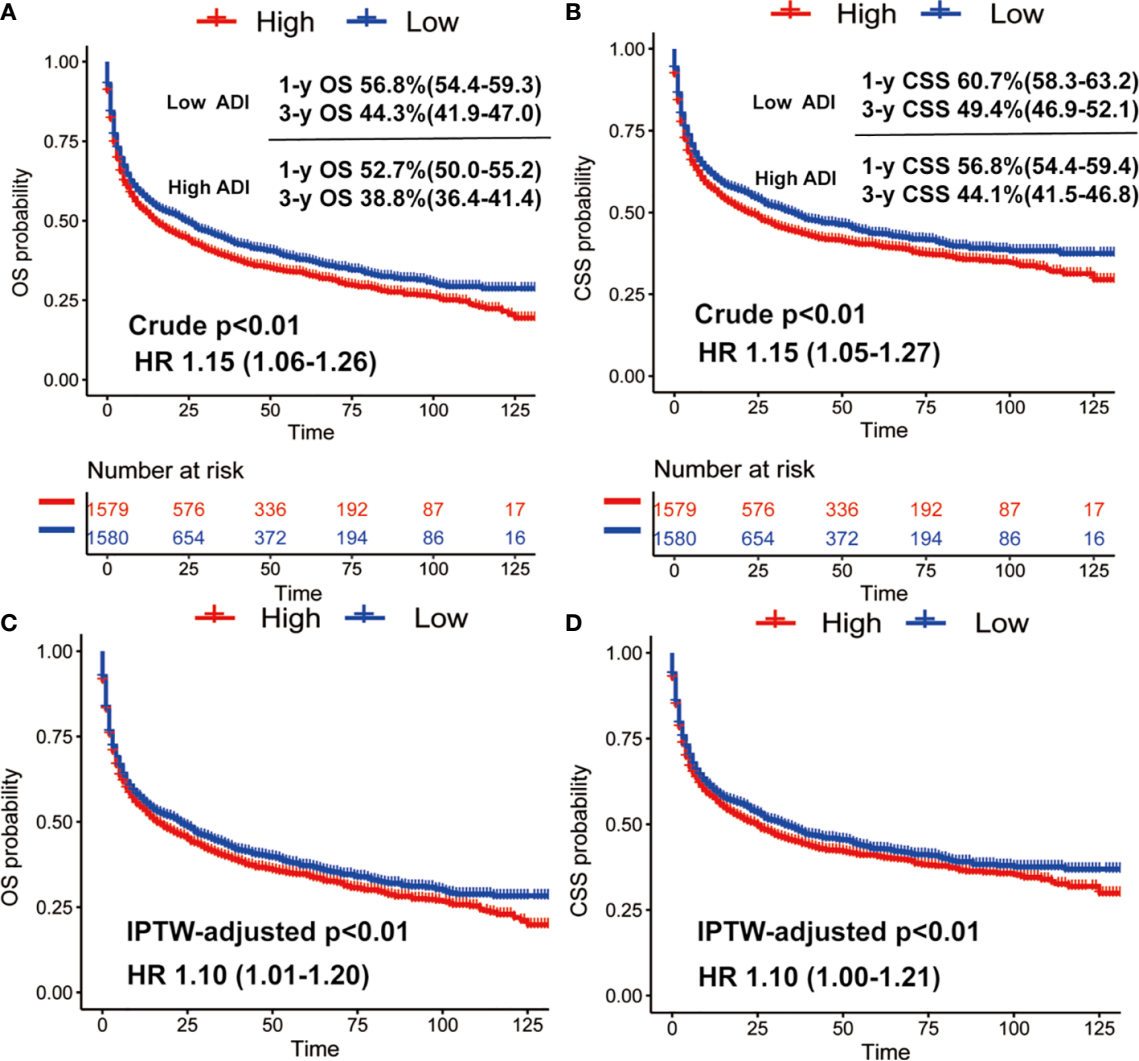
Crude Kaplan-Meier survival curves stratified by ADI for overall survival **(A)** and cancer-specific survival **(B)**; IPW-adjusted Kaplan-Meier survival curves stratified by ADI for overall survival **(C)** and cancer-specific **(D)**.

**Figure 2:**
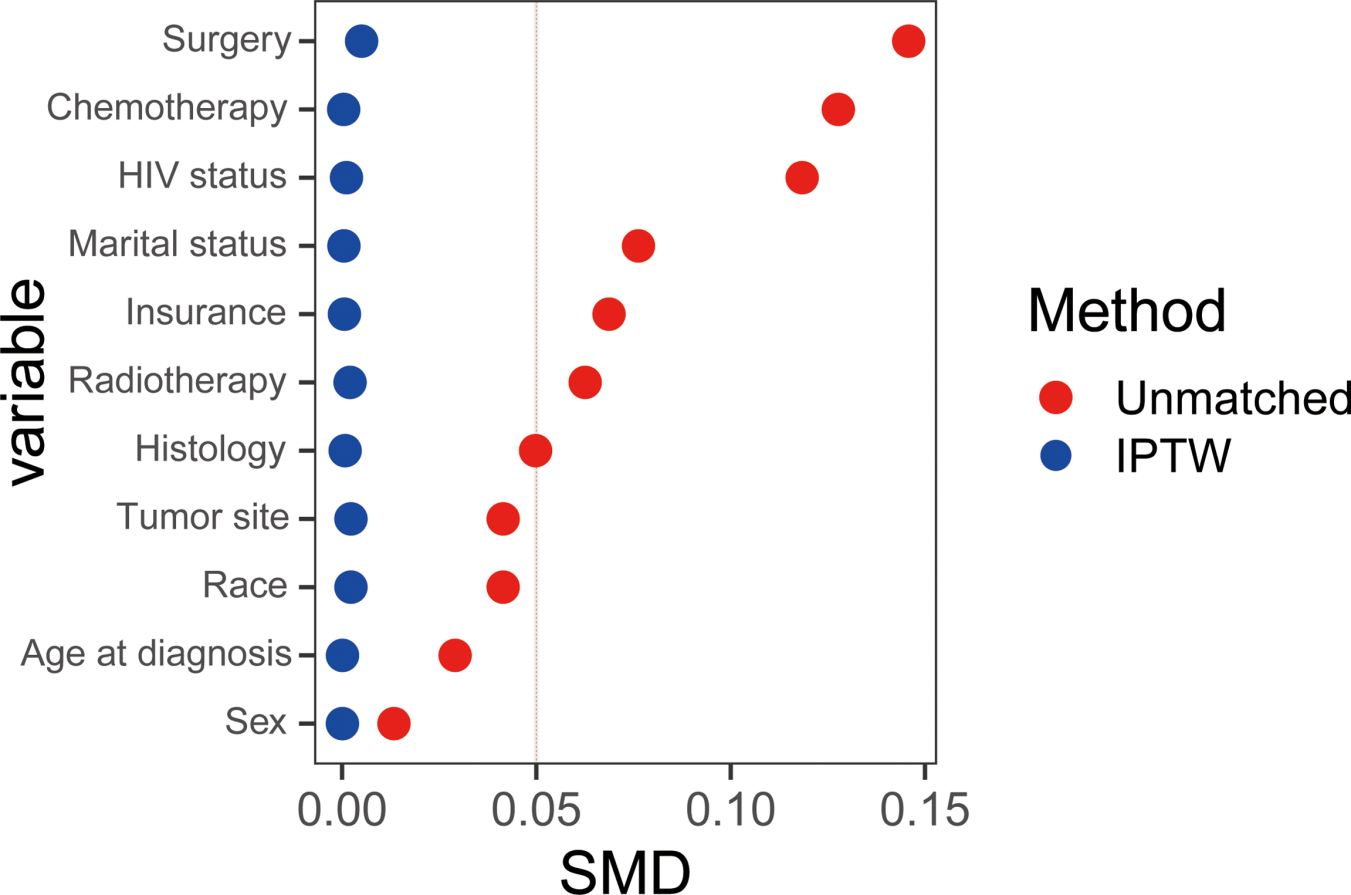
The absolute Standardized Mean Differences (SMDs) were calculated to verify the covariate balances after the IPW adjustment and a difference of SMD equal to zero indicates ideal balance.

We then conducted Kaplan-Meier survival analysis of OS and CSS for low and high ADI patients. As shown **Figure 1**, in crude KM analysis, low ADI was significantly associated with higher OS rates (HR 1.15, 95%CI 1.06-1.26, P<0.01). The 1- and 3-year OS were 56.8% (95%CI, 54.4%-59.3%) and 44.3% (95%CI, 41.9%-47.0%) in low ADI cohort, whereas the corresponding OS were 52.7% (95%CI, 50.0%-55.2%) and 38.8% (95%CI, 36.4%-41.4%) in high ADI patients. In terms of endpoint of CSS, similar result with higher CSS rates in low ADI patients was also observed (HR 1.15, 95%CI 1.05-1.27, P<0.01). And the 1-year and 3-year CSS were 60.7% (95%CI, 58.3%-63.2%) and 49.4% (95%CI, 46.9%-52.1%) in low ADI group, whereas the corresponding CSS were 56.8% (95%CI, 54.4%-59.4%) and 44.1% (95%CI, 41.5%-46.8%) in high ADI group. To further intensify our findings, we additionally performed IPW analysis to adjust the potential confounding. Excellent balances between the two ADI groups were achieved regarding all covariates (**Figure 2**). And IPW-adjusted survival analysis showed that low ADI still demonstrated better OS and CSS [OS: IPTW-adjusted HR 1.10, 95%CI 1.01-1.20, P<0.01; CSS: IPTW-adjusted HR 1.10, 95%CI 1.00-1.21, P<0.01; **Figure 1**]. Multivariable Cox proportional hazards regression further revealed increased adjusted overall mortality (HR 1.10, 95%CI 1.01-1.20) and cancer-specific mortality (HR1.10, 95%CI 1.00-1.21) for high ADI patients.

### Subgroup analysis

**Figure 3:**
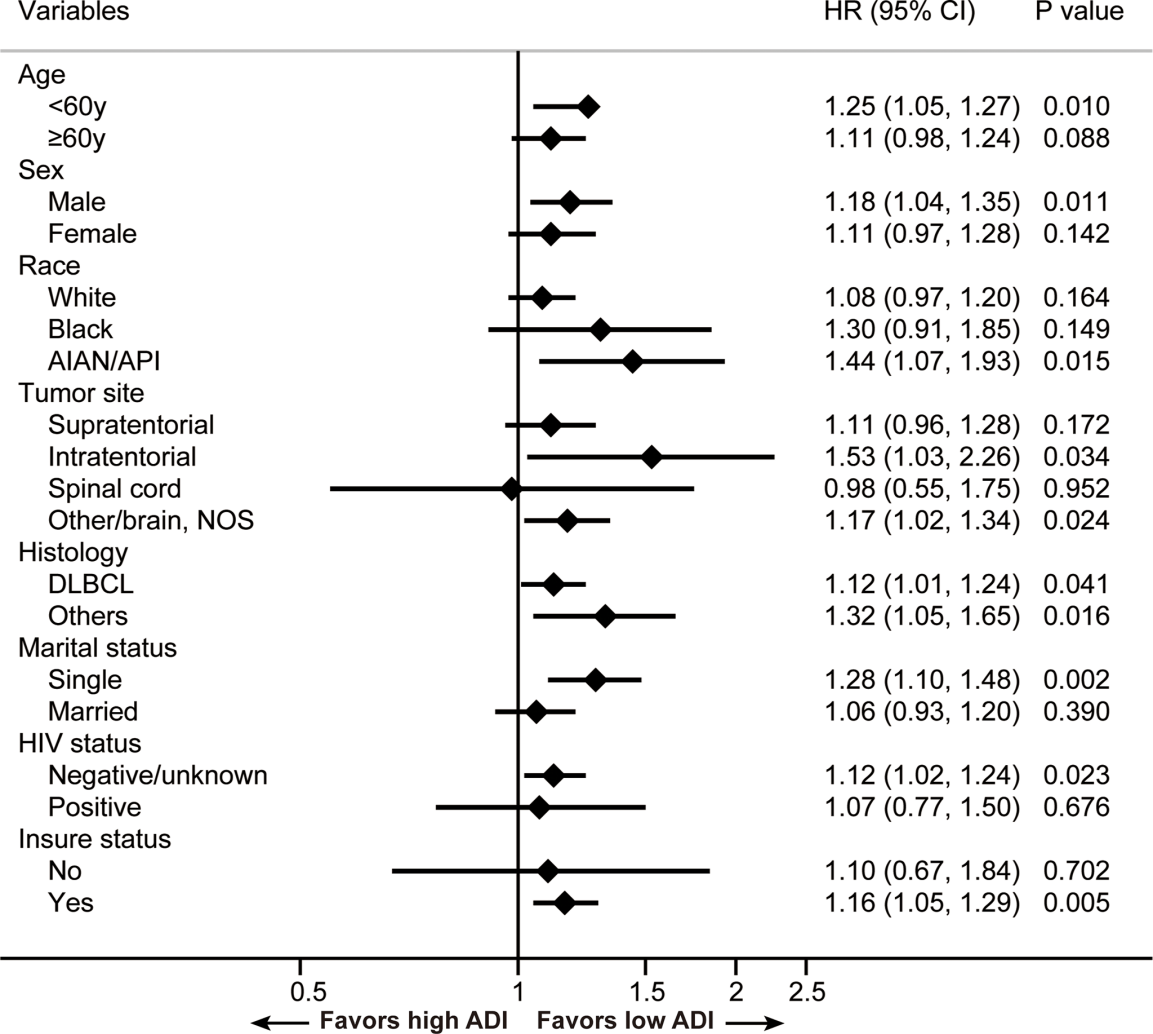
Results for the subgroup analyses for cancer-specific survival are summarized in a forest plot. AIAN/API, American Indian/Alaska Native or Asian Pacific Islander; DLBCL, diffuse large B-cell lymphoma; NOS, not otherwise specified.

To explore the relation between ADI and CSS in different patient subsets, subgroup analyses were conducted and results are summarized in **Figure 3**. We found ADI could remain a prognostic indictor among subgroups. And ADI demonstrated more relatively robust in patients who were less than 60 years old, male, AIAN/API, with infratentorial tumors, with other PCNSL subtypes, single, with negative HIV status and insured.

### Multiple mediation analysis

**Figure 4:**
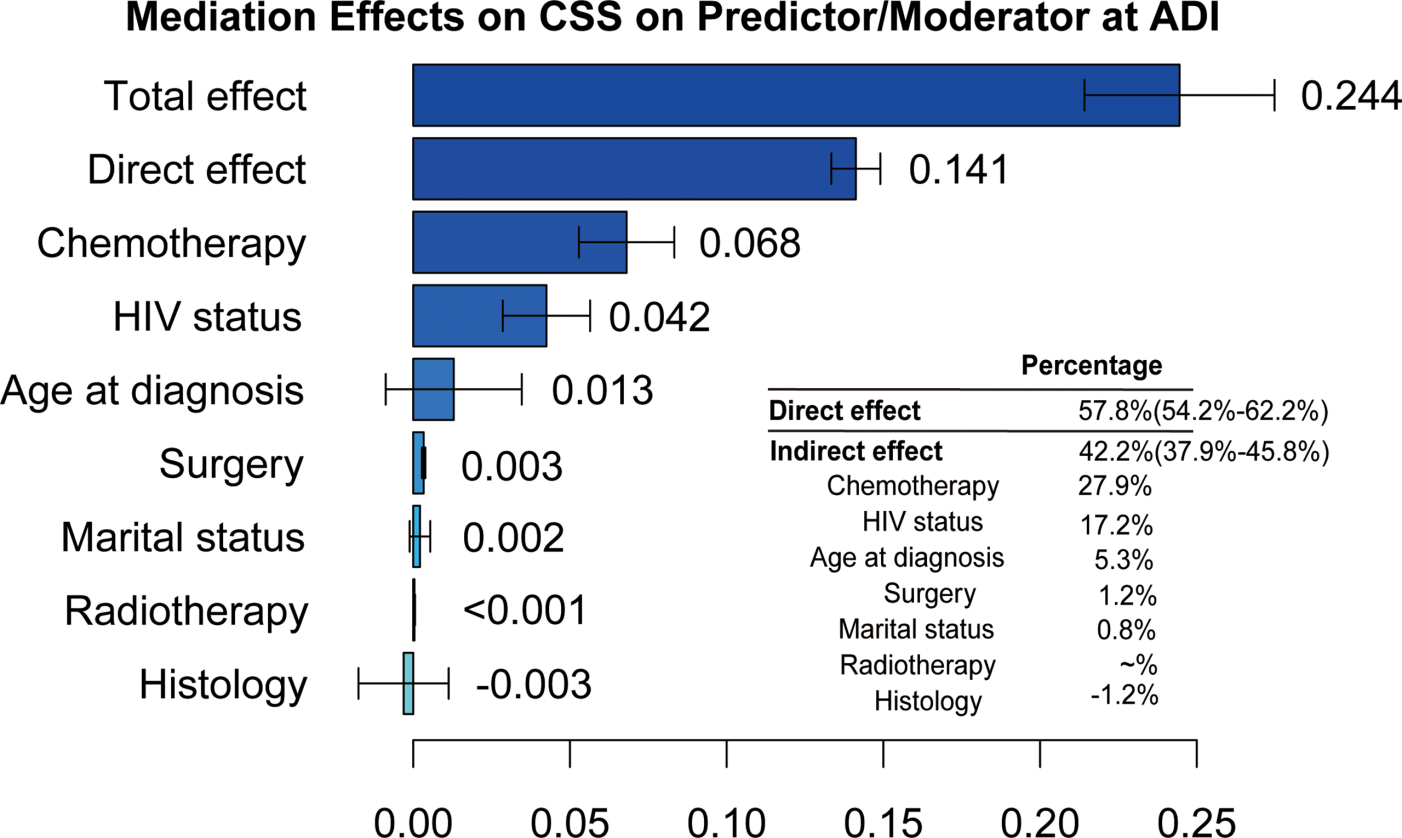
The estimation of direct and indirect effects contributing to the disparities related to the ADI on cancer-specific survival in PCNSL patients.

Subsequently, we further performed MMA to investigate the contribution of estimated direct and indirect effects to the CSS disparities between two ADI subgroups. As illustrated in **Figure 4**, we found estimated direct effect that mediating factors failed to account for was 57.8% (95%CI, 54.2%-62.2%), whereas indirect effect was 42.2% (95%CI, 38.9%-45.8%) where the chemotherapy (27.9%; 95%CI, 21.3%-34.0%) made a greatest contribution, followed by HIV status (17.2%, 95%CI, 11.5%-23.0%).

### Association of ADI with chemotherapy application

Chemotherapy is the main treatment for PCNSL patients, and almost 70% patients initiated some form of chemotherapy in our cohort. Finally, we examined the extent to which area-based socioeconomic deprivation as well as other sociodemographic variables predicted receipt of chemotherapy. In multivariable logistic regression, ADI was found to be significantly related to the odds of initiating chemotherapy (odds ratio of 0.82 for the high ADI compared with the low ADI; 95% CI 0.70-0.97, P=0.018). Moreover, older age, black race, other PCNLS subtypes, single status, positive HIV status and no insured were observed to be significantly associated with less use of chemotherapy (all P<0.05, **Table 2**).

**Table 2:** Multivariable logistic regression to predict odds of receiving chemotherapy for PCNSL patients.

Variables	OR	95%CI	*P*
Age			
<60y	Ref		
≥60y	0.5	0.42-0.60	<0.001
Sex			
Male	Ref		
Female	1.06	0.90-1.25	0.484
Race			
White	Ref		
Black	0.50	0.38-0.66	<0.001
AIAN/API	0.94	0.73-1.21	0.651
Tumor site			
Supratentorial	Ref		
Infratentorial	0.73	0.52-1.03	0.071
Spinal cord	1.2	0.83-1.73	0.336
Other/brain, NOS	0.98	0.82-1.16	0.766
Histology			
DLBCL	Ref		
Others	0.53	0.44-0.64	<0.001
Marital status			
Single	Ref		
Married	1.52	1.28-1.81	<0.001
HIV status			
Negative/unknown	Ref		
Positive	0.28	0.19-0.40	<0.001
Insurance status			
No	Ref		
Yes	1.54	1.02-2.34	0.041
ADI			
Low	Ref		
High	0.82	0.70-0.97	0.018

AIAN/API, American Indian/Alaska Native or Asian Pacific Islander; DLBCL, diffuse large B-cell lymphoma; NOS, not otherwise specified; STR, subtotal resection; GTR, gross total resection; ADI, area deprivation index.

## Discussion

The results of our national, population-based study showed that neighborhood SES, as measured by ADI, was independently associated with both OS and CSS in PCNSL. And the survival disparities remained stable after adjusting for multiple factors *via* IPW analysis and multivariable Cox proportional hazards regression analysis. Furthermore, MMA revealed that several factors including chemotherapy and HIV status making up about 40% of the overall effect, mediated PCNSL survival disparities related to the ADI. These findings add to the growing literatures uncovering the impact of disadvantaged neighborhood SES on cancer outcomes.

To our knowledge, this is the first study to focus on the impact of the neighborhood SES, measured by ADI, on PCNSL survival. Instead of applying single-metric or one-off composite neighborhood SES features, our study complemented prior works *via* employing a comprehensive composite measure to evaluate the complexity of community environment. Socioeconomic deprivation has been observed to be in connection with increased cancer incidence, lack of treatments and inferior outcomes across multiple tumors (15, 20, 27). Yu et al. reported that ADI level partially accounted for the tumor characteristics at presentation and survival disparities in colorectal cancers, and they further analyzed mediating factors to gain deeper understand on neighborhood SES (28). Also, our study broadens the type of tumors affected by the neighborhood SES, and neighborhood disadvantage was found to play an important role in survival of these highly malignant tumors.

Through MMA, the application of chemotherapy was observed to mediate the greatest share of ADI disparity on PCNSL survival. Approximately one-fourth of the ADI disparity on PCNSL survival was attributed to differences in the receipt of chemotherapy. High-dose methotrexate-based chemotherapy has demonstrated high efficacy and it has greatly improved PCNSL outcomes. And omission of chemotherapy was found to be closely related to indicators of poor socioeconomic status (14). Consistently, our study also confirmed that ADI was significantly associated with the odds of initiating chemotherapy. These may partially explain MMA results. Meanwhile, multivariable logistic regression showed other factors including age, race, marital status, insurance status and HIV status were independently related to receipt of chemotherapy. The disparities in application of chemotherapy for PCNSL highlights the need to improve delivery of systemic treatment in the community setting. And In-depth researches are needed to explore the internal connection.

Furthermore, HIV status was observed to be associated with 17.2% of the effect of ADI. Many studies have demonstrated that HIV diagnosis rates are higher for individuals from low-SES communities compared with those from high-SES communities (29, 30). Also, once infected with HIV, persons with acquired immune deficiency syndrome (AIDS) had a markedly increased risk of malignancies including PCNSLs (31). Given the comorbidity and poor performance status of HIV-related PCNSL patients, as well as the association between positive HIV status and the nonreceipt of chemotherapy which was also found in our analysis, they usually have a worse prognosis relative to counterparts with normal immune function, indicating the importance of HIV prevention and treatment. Therefore, through a mediation model, we demonstrated that policies development to enhance health delivery at the community level is a vital step to improve equity on PCNSL prognosis. And health policy makers and medical institutions should also take multilevel initiatives to strengthen HIV prevention and treatment.

Moreover, multiple factors have been found to account for cancer survival inequality in deprived neighborhoods including limited access to healthcare resources, lack of socioeconomic support, and barriers to travel for initial and follow-up care (20, 32, 33). Although SES information at the individual level may be more accurate and indicative, county-based socioeconomic deprivation has been reported to be associated with nonoptimal treatment and inferior survival independently of individual SES (20); and it can offer insight into cultural and group-level phenomena, may exerting more guiding effect on the implementation of macro medical policy. Besides, Unger et al. reported that association of high area-level socioeconomic deprivation with worse cancer outcomes persisted in clinical trials where cancer patients get access to protocol-directed care (34), suggesting that neighborhood SES should be cautiously considered for researchers to design and interpret clinical trials. The findings of our analysis may provide some meaningful implications for PCNSL-related clinical trial design and healthcare policy. Future studies are also needed to explore the role of ADI in other type of tumors to support health policy interventions at the community level.

There are some several limitations should be acknowledged. Although ADI has excellent quantitative ability for neighborhood socioeconomic disadvantage which had been validated in some articles (20, 28), it cannot reflect every aspect of neighborhood SES. Due to inherent limitations of the SEER database, some prognostic factors such as comorbidities, Karnofsky performance status, and specific therapeutical program, as well as individual SES were failed to be adjusted. Moreover, the findings of this study are based on the SEER database, and whether they can be generalized to other groups needs further verification. In spite of these shortcomings mentioned above, the methods applied in our analyses were rigorously designed and the results still offer certain referential value.

## Conclusions

The current study found that ADI was significantly associated with receipt of treatment and cancer prognosis in PCNSL patients. And several factors including chemotherapy and HIV status of PCNSL patents contributed to the CSS disparities between ADI subgroups were uncovered by MMA. Such relationships would highlight the importance of policies development to enhance healthcare delivery and promote awareness of HIV prevention and treatment in low-resource neighborhoods. And this study also supports policies for ongoing investments in low-SES communities. Policymakers and payers should take socioeconomic deprivation into consideration to maximize the efficiency and potency of healthcare strategies. Furthermore, individual-level SES is underexamined in our study given the inaccessibility of data of individual-level SES in SEER database, future studies need#146;to investigate contribution of individual patient-level SES to PCNSL survival, and to better assess cancer outcomes in the context of both neighborhood and individual socioeconomic disadvantage.

## Data availability statement

Publicly available datasets were analyzed in this study. This data can be found here: https://seer.cancer.gov/data-software/.

## Author contributions

XD designed the study; XD, XY, and CY contributed to data analysis. XD wrote the initial draft of the manuscript; XD, XY, CY, KC, JuZ, QZ, TL, QT, and JiZ reviewed and edited the manuscript. All authors read and approved the manuscript.

## Funding

This work was supported by grants (2018YFA0107900, 92168103, 32171417, 2019CXJQ01), from Ministry of Science and Technology of China, National Nature Science Foundation and Shanghai Municipal Government, Peak Disciplines (Type IV) of Institutions of Higher Leaning in Shanghai.

## Acknowledgments

The authors would like to thank the SEER database for the availability of the data.

## Conflict of interest

The authors declare that the research was conducted in the absence of any commercial or financial relationships that could be construed as a potential conflict of interest.

## Publisher’s note

All claims expressed in this article are solely those of the authors and do not necessarily represent those of their affiliated organizations, or those of the publisher, the editors and the reviewers. Any product that may be evaluated in this article, or claim that may be made by its manufacturer, is not guaranteed or endorsed by the publisher.
